# A survey of neurophysiological differentiation across mouse visual brain areas and timescales

**DOI:** 10.3389/fncom.2023.1040629

**Published:** 2023-03-13

**Authors:** Saurabh R. Gandhi, William G. P. Mayner, William Marshall, Yazan N. Billeh, Corbett Bennett, Samuel D. Gale, Chris Mochizuki, Joshua H. Siegle, Shawn Olsen, Giulio Tononi, Christof Koch, Anton Arkhipov

**Affiliations:** ^1^MindScope Program, Allen Institute, Seattle, WA, United States; ^2^Department of Psychiatry, University of Wisconsin–Madison, Madison, WI, United States; ^3^Department of Mathematics and Statistics, Brock University, St. Catharines, ON, Canada

**Keywords:** visual cortex, neurophysiological differentiation, mouse, Allen Institute for Brain Science, Neuropixels, conscious perception

## Abstract

Neurophysiological differentiation (ND), a measure of the number of distinct activity states that a neural population visits over a time interval, has been used as a correlate of meaningfulness or subjective perception of visual stimuli. ND has largely been studied in non-invasive human whole-brain recordings where spatial resolution is limited. However, it is likely that perception is supported by discrete neuronal populations rather than the whole brain. Therefore, here we use Neuropixels recordings from the mouse brain to characterize the ND metric across a wide range of temporal scales, within neural populations recorded at single-cell resolution in localized regions. Using the spiking activity of thousands of simultaneously recorded neurons spanning 6 visual cortical areas and the visual thalamus, we show that the ND of stimulus-evoked activity of the entire visual cortex is higher for naturalistic stimuli relative to artificial ones. This finding holds in most individual areas throughout the visual hierarchy. Moreover, for animals performing an image change detection task, ND of the entire visual cortex (though not individual areas) is higher for successful detection compared to failed trials, consistent with the assumed perception of the stimulus. Together, these results suggest that ND computed on cellular-level neural recordings is a useful tool highlighting cell populations that may be involved in subjective perception.

## Author summary

Information about visual stimuli is well-known to be represented across several brain regions. However, information may be available and yet not subjectively perceived. Since percepts are determined by neural activity, the number of distinct percepts experienced over a period of time must be reflected in the number of distinct activity states the brain region occupies in that time, called *neurophysiological differentiation* (ND). ND of the entire brain has been shown to reflect subjective reports of stimulus meaningfulness. But which specific populations of neurons within the brain support conscious visual perception, and what is the correct timescale at which states should be quantified? We address these questions by analyzing ND of spiking activity in the mouse visual system.

## Introduction

A key requirement for understanding the mechanistic origin of subjective, conscious visual perception is the ability to quantify visual experience based on neural activity. In the neuroscience of vision, for instance, regions of the primate brain that reflect information content of visual stimuli, such as edges, objects, faces, etc. have been identified and extensively characterized (Rolls, 2000). But availability of information does not imply that it is necessarily utilized by the brain, much less that it is subjectively perceived (Shimojo et al., 2001; Koch, 2004; Brette, 2019; Buzsáki, 2019).

**FIGURE 1:**
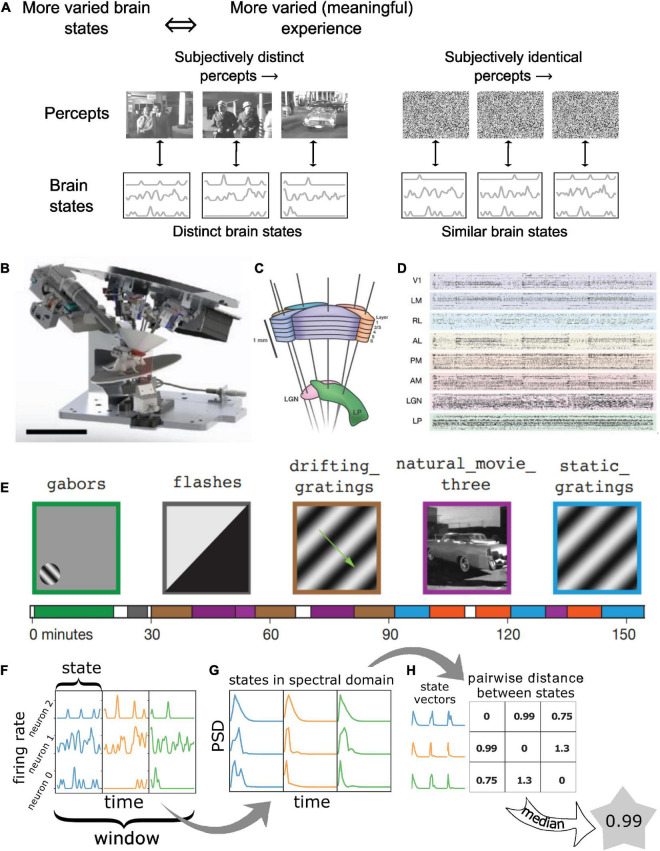
Quantifying “meaningfulness” of subjective percepts using neurophysiological differentiation. **(A)** Distinct subjective percepts must correspond to distinct physiological ‘states’ of the brain or the part of it that is the substrate for the experience. This can be captured by quantifying the size of state space explored by the appropriate brain region over a period of time. **(B)** Schematic of the experiment. Mice are head-fixed and free to run on a rotating platform. Visual stimuli are presented on a screen and responses are measured using 6 Neuropixels probes **(C)**. **(D)** Spiking activity simultaneously recorded from 6 visual cortical regions as well as thalamus and hippocampus were used for the analysis. **(E)** Different classes of stimuli were presented including a control no-stimulus condition, simple artificial stimuli such as Gabor patches and full field flashes, more complex artificial stimuli such as static and drifting gratings and natural movies along with their time-shuffled versions. These stimuli span a wide range of stimulus complexity. Panels **(B–E)** adopted with permission from Siegle et al. (2021). **(F–H)** The number of brain states are quantified using the neurophysiological differentiation (ND) metric. Spikes are binned and convolved with a Gaussian; the resulting firing rate timeseries are divided into windows which are subdivided into states **(F)**; states are quantified in terms of the PSD of each neuron’s activity within each state concatenated into a single state vector **(G)**; pairwise Euclidean distance is calculated between all states within a window; the median distance for a window is defined as the ND for the window **(H)**.

Consider, for instance, a conscious human observer watching a meaningful movie versus viewing an analog television displaying white noise or “snow” (Figure 1A). Both stimuli are constantly changing in time and have high diversity in space, so that, at the level of pixels on the screen, they both represent complex and highly dynamic patterns. However, for TV noise, the perceptual experience of an observer is low in complexity and remains approximately constant over time (TV noise is simply perceived as a more-or-less homogeneous, “noisy pattern” from moment to moment). In contrast, almost any scene from an engaging movie might change slowly, thus having lower temporal complexity, but is nonetheless more meaningful to the viewer, evoking distinct visual percepts over time. Since percepts are determined by neural activity, each specific percept must correspond to a specific pattern of activity in the neural population that supports subjective visual perception, with a one-to-one mapping between the two (Boly et al., 2015).

In the example above, we would expect TV noise to result in relatively stable activity corresponding to the unchanging percept, whereas the movie scene would evoke temporally varying activity corresponding to each of the distinct percepts. Note that this will not be universally true for any neural population (for instance, it will not be true for retinal photoreceptors); here we refer only to a population that specifically supports subjective visual perception. Which neurons in the brain constitute such a population remains unknown at present. Thus, the richness of perceptual experience (“meaningfulness of stimuli”) should correspond to the richness of neural activity (and not the richness of the stimulus) in the specific neuronal populations that are the physical substrate of the perceptual experience. This richness of neural activity has been quantified in several recent studies by measuring how many different states the brain or a specific brain region enters during a period of time, called *neurophysiological differentiation* (ND) (Synek, 1988; Gosseries et al., 2011; Barttfeld et al., 2015; Boly et al., 2015; Hudetz et al., 2015; Solovey et al., 2015; Mayner et al., 2022).

A few different metrics of ND have been proposed to infer the meaningfulness of visual stimuli to humans, such as Lempel-Ziv complexity (Boly et al., 2015) or spectral differentiation (Mensen et al., 2017, 2018). Some of these metrics correlate with subjective reports from participants as to the “interestingness,” “understandability,” or “meaningfulness” of the visual stimuli (Mensen et al., 2018). However, differentiation can be probed only at coarse spatial resolutions in human studies. To further our understanding of the relationship between differentiation of activity in specific brain regions and “meaningfulness” of stimuli, we turn to studying differentiation at the cellular level in the mouse brain, leveraging readily accessible high-resolution and high-throughput recording techniques.

A recent calcium imaging study showed that the ND of cellular fluorescent responses was higher for naturalistic stimuli (movies of predators and prey) compared to phase-scrambled versions of the same movies in in layer 2/3 of two specific regions of the mouse brain, VISal and VISam. In layers 4 and 5 of the same areas and in all layers of the other areas studied (VISp, VISl, and VISpm), the difference was non-significant (Mayner et al., 2022). In contrast, activity in any recorded visual cortical layer and region could be used to accurately decode stimulus type (naturalistic vs. scrambled). While not a conclusive measure of perceived experience, this showed that the metric of ND used in this study, spectral differentiation, was thus able to indicate with high specificity mouse brain regions that are potentially involved in perception as opposed to merely representing stimulus information. Here, we apply a similar analysis to Neuropixels recordings from the mouse brain, allowing us to probe for the first time the spectral differentiation of spiking activity across a wider range of spatiotemporal scales.

We first analyze the dependence of ND on the timescale of observation for single neurons as well as ensembles. We find that the ND of activity of single neurons does have an optimal timescale, close to the autocorrelation time of their firing rate (∼100 ms); but this optimal timescale is shifted to <5 ms for ensembles of neurons. We then fix the timescale and vary the composition of ensembles to understand how ND of response to different stimuli behaves in different regions of the mouse visual hierarchy. We find that in most (though not all) visual cortical areas, ND is higher for naturalistic stimuli compared to artificial ones. On the other hand, the ordering of response ND does not necessarily follow the ordering of the spectral differentiation of the stimulus itself (stimulus differentiation, or SD in short), suggesting that our metric is capturing more than just the information content of stimuli. We also show that depending on the behavioral state of the animal (whether it is running or stationary), ND of activity in individual cortical layers may or may not be modulated by the stimuli.

We repeat these analyses for mice performing a visual image change detection task to test the metric against a behavioral correlate of perception. ND of activity of the entire visual cortex is higher for successful trials (hits) compared to failures (misses), though the difference is not significant in individual areas. These differences are also modulated by the behavioral state (running vs. resting) of the animal.

Our results demonstrate that the spectral differentiation metric applied to high resolution electrophysiological recordings can identify specific neural populations that appear to be sensitive to the expected perceptual differences in stimuli and correlates with a behavioral readout of perception. In contrast, a simpler metric of variation in brain states, the variance of mean firing rate across states, does not correlate with the expected perceptual differences in most neural populations that we analyzed.

## Materials and methods

### Computing spectral differentiation

Spectral differentiation is computed on timeseries data. Therefore, for each neuron, spike times are converted into a binary timeseries with a resolution of 5 ms bins (equivalent to a sampling rate of 200 Hz), depending on the presence or absence of a spike in each bin. This binary timeseries is then convolved with a normalized Gaussian kernel with a halfwidth of 2 bins (10 ms) and cut off on either side at 5 bins (25 ms) to obtain the smoothened firing rates. Note that on an average less than 2% of spikes for any neuron occur within 5 ms of another spike; thus, we do not expect the binarization process to lead to any significant loss of information. The ND of only ∼2% windows at most could be slightly altered.

Spectral differentiation is defined for an ensemble of *N* neurons over windows of size *W*, for states with state length *S* (*W/S* state intervals in total per window; Figure 1F). For each state interval, the “spectral state” of that interval is quantified in terms of the power spectrum density (PSD) of the signal of each neuron within the state window: for this, the spectra are first computed using the NumPy function *numpy.fft.rfftn* (this function computes the N-dimensional discrete Fourier transform over any number of axes in an M-dimensional real-valued array) and the absolute value is squared to obtain the power spectrum (Figure 1G). Since the sampling frequency is fixed at 200 Hz, power spectrum is obtained over frequencies ranging from –100 to 100 Hz with a resolution of 1/*S* Hz. We only keep non-negative frequencies in the power spectrum since the FFT is symmetric. The power spectra for each neuron are concatenated into a (100 *N S*) dimensional vector (Figure 1H). Euclidean distance is computed between all pairs of such *W / S* vectors and the median distance is defined as the spectral differentiation (Figure 1H).

To investigate the potential boundary effects while computing PSD due to small state sizes, we repeated the analysis using Tukey tapering. In this case, PSD for each state segment of the timeseries was computed using the SciPy function *scipy.signal.spectrogram*, with the Tukey shape parameter set to 1. Our results do not change with the use of tapering (Supplementary Figure 3-4).

We also repeat the analysis for a simpler metric of variation across states: the variance of mean firing rate across states, called “mean firing rate differentiation.” This metric is computed as follows: first we normalize the firing rate of all neurons in an ensemble by dividing by their mean firing rate through the entire session. Next, we use the normalized firing rates to compute the mean firing rate across all neurons in each state (*S*), and then compute its variance across all states within a window of time (*W*).

All differentiation metrics are computed in areas that have at least 10 neurons, to avoid artifacts due to singular extreme neurons.

### Normalization of spectral differentiation

Spectral differentiation is a non-negative quantity, but does not have an upper bound (see section “Materials and methods”). Therefore, the absolute value of the measure is not directly meaningful. The relative magnitude of the metric across different stimuli, or across different ensembles of neurons is of primary interest.

To enable comparison of the metric across different neural ensembles, several normalizations are performed. First, note that scaling all firing rates by a constant factor Q changes the PSD by a factor of Q^2^; thereby all distances in the state space change by Q^2^; thus ND changes by a factor of Q^2^, without altering the temporal complexity of the signal. Thus, to account for the differences in the mean firing rates, the firing rates are divided by the ensemble mean firing rate before computing differentiation.

Second, as the number of neurons increases, the dimensionality of the state space in which distances are computed also increases. To account for the variations in the number of neurons across experiments and brain regions, we normalize by an additional factor of *sqrt(N)*, where *N* is the number of neurons. The state of the neural ensemble is defined by concatenating the PSD of *N* neurons together, giving a *N* × *len(PSD)* dimensional representation. Assuming that each dimension contributes approximately equally to the spread in the states, the mean distance is thus expected to increase with *N* as *sqrt(N)*. We verify this normalization by computing ND of activity of several subpopulations of a sample population of neurons. We find that ND indeed increases as the square root of the fraction of neurons included in the subpopulation (Supplementary Figure 1-1).

The state length also affects the dimensionality of the final state vector, which is given by 100 *N S*. However, unlike neurons, the dimensions contributed by different frequencies in the PSD do not contribute equally to the distances between states. The mean (0-frequency) component of the PSD is the primary contributor to the distance between states, and thus we do not normalize for dependence of dimensionality on state length. However, the total power in FFT scales as the square of the number of samples, i.e., state length, so differentiation is normalized by dividing by *S*^2^ (see section “Bounds on spectral differentiation” for further clarification).

Overall, we have


(1) $$\text{ND}={\text{ND}}_{\text{raw}}\,\left(\text{FR}/\text{mean}\,\left(\text{FR}\right)\right)\;N^{-0.5}\;S^{-2}$$

Where ND_raw_(⋅) is as described in the preceding section (computing spectral differentiation) and FR is the vector of time-binned firing rates for all neurons in the analyzed ensemble.

### Bounds on spectral differentiation

Spectral differentiation has a lower bound of 0, since it is the median distance between states. For the upper bound:

Each Fourier component is bounded by the length of the signal (number of samples, *fS*) times the maximum amplitude of the signal (*A*):


(2) x⁢[k]≤A⁢f⁢S

where the sampling frequency, f, is set at 200 Hz, and S is the state length in seconds. *A* here has units of firing rate (# spikes / second). Each element in the PSD is thus bounded above by:


(3) $$P\,S\,D\,\left(k\right)\leq A^{2}\,f^{2}\,S^{2}$$

Consequently, since we have N neurons, the median distance is bounded above by:


(4) $$N\,D\leq \sqrt{N}\,A^{2}\,f^{2}\,S^{2}$$

We normalize differentiation by dividing by $\sqrt{N}$, and by *S*^2^, after which the differentiation becomes bounded above by:


(5) $$N\,D\leq A^{2}\,f^{2}$$

where *A*, as mentioned earlier, is the maximum amplitude of the signal (firing rate) over the time window under consideration. Now to account for the variations in mean firing rates across ensembles, we divide the firing rate timeseries of each unit by the mean firing rate across all units in the ensemble (restricted to RS or FS units, whichever we are computing ND for), and across the entire recording time; and then compute differentiation of that timeseries. In principle, this can lead the normalized firing rate to be indefinitely large (i.e. *A* is unbounded above). Thus, ND is strictly speaking unbounded. This is of course a degenerate case, and has no physical relevance. It is saying that because there is a single spike in a very long timeseries, that one state is infinitely far away from all other states.

In practice, we observe that the firing rates divided by the mean ensemble firing rate, are typically less than ∼50. Therefore in practice, ND values should be less than (50 200*s*^−1^)^2^ = 10^8^*s*^−2^. Indeed, our observed ND values are typically under ∼3×10^6^*s*^−2^, well within this upper bound.

### Characteristic timescale of stimuli and neural activity

**FIGURE 2:**
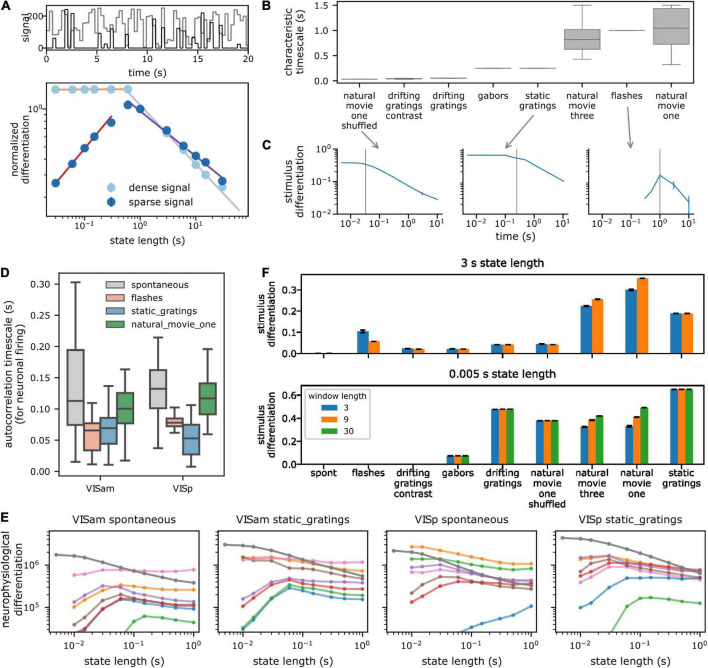
Dependence of spectral differentiation on the temporal scale of observation. **(A)** Top toy example of a 1D timeseries with characteristic timescale (CT) of 600 ms (light: dense and dark: sparse signal). **(A)** Bottom spectral differentiation of above signals as a function of state length S. Colored lines show power law fits. For S > CT, differentiation decreases as a power law; for shorter S, differentiation remains constant for dense (light) but decreases for sparse signals (dark). **(B)** CT of pixel values in the visual stimuli. Box plot shows distribution across all pixels. Vertical gray line indicates median CT from panel **(B)**. **(C)** Stimulus differentiation (SD) vs. S for three stimuli. SD peaks (sparse signal) or plateaus (dense signal) around the CT of the signals (gray lines). Error bars are standard deviation. **(D)** CT of a subsample of neurons from a single session for responses to different stimuli **(E)**. Neurophysiological differentiation (ND) of firing rate of individual neurons (colored lines) from two example areas and for two stimuli. Individual neurons have an optimal time at which ND peaks. Optimality shifts to timescale <5 ms when ND is computed for all neurons from the respective areas (dark gray line). Error bars are standard error of mean (SEM). Different colors indicate different neurons. **(F)** SD for two extreme state lengths (top and bottom), for three example values of window lengths W in s (colors) for all stimuli. SD is insensitive to W, but depends very sensitively on S, the timescale of observation (e.g., static gratings have the highest SD for S = 5 ms but movies have a higher SD for S = 3 s). Error bars are standard deviation.

The “Characteristic timescale” (CT) of a signal is the timescale over which it remains constant before changing substantially. For piecewise constant uncorrelated signals such as the toy example of Figure 2A (600 ms), or stimuli like Gabors, static gratings (250 ms), and shuffled movies (33 ms), which change after a fixed period and take random values, the CT is simply the time for which the signal remains constant before changing. The Flashes stimulus remains gray for 1.75 s and turns black or white for 0.25 s, and thus has a characteristic timescale of 1 s on average over which it remains constant.

For all other signals, the characteristic timescale is computed as the timescale over which the signal’s autocorrelation decays exponentially. To obtain autocorrelation timescales (AC), autocorrelation of firing rates (or pixel intensities for stimulus AC) are first computed. The autocorrelation is best modeled by two exponents, over an initial fast timescale and a slower timescale, with the transition point between the timescales changing from neuron to neuron. To account for this, the logarithm of the autocorrelation is fit using *scipy.curvefit* to:


(6) $$f\left(t\right)=-a-x/t_{1}\;\mathrm{for}\left(x<c\right)$$

(7) $$f\left(t\right)=\left(x-c\right)/t_{2}+\left(-a-c\right)/t_{1}\mathrm{for}\left(x\geq c\right)$$

Parameters *a*, *c*, *t_1_*, *t_2_* are fit to data. *t_1_* is constrained between 0 and 1.5 s; *t_2_* is constrained between 0.5 and 20 s; and *c* between 0.03 and 0.2 s. We only consider the fast timescale *t_1_* for all other analysis.

Note that for piecewise constant signals, the autocorrelation does not give a good estimate of the timescale over which the signal typically remains constant.

### Code availability

All code required for the analysis presented in this article is available on GitHub at https://github.com/gsaurabhr/npx_differentiation.

## Results

For the analysis presented here, we use the Visual Coding Neuropixels dataset (Siegle et al., 2021) published by the Allen Institute at www.brain-map.org; both the Brain Observatory and Functional Connectivity stimulus sets were included. In these experiments, mice were head-fixed but otherwise free to run on a rotating disk, while visual stimuli were displayed on a screen in front of them (Figure 1B). Action potentials were recorded simultaneously from ∼6 ± 1 Neuropixels electrodes with 384 channels each, sampled at 30 kHz, covering broad regions of the mouse cortical visual hierarchy (the primary visual cortex or area VISp, and the higher visual areas VISl, VISrl, VISal, VISpm, VISam; Figure 1C) as well as thalamic regions (LGd, LP) and hippocampus. The dataset consists of spiking activity for 17,129 regular spiking (RS; mean waveform width > 0.4 ms) and 3,910 fast spiking (FS; mean waveform width < 0.4 ms) neurons with SNR > 2.5 across 58 recording sessions (distinct mice) and spanning the above-mentioned areas (∼2,121 ± 663 RS and 470 ± 183 FS neurons per cortical area on average) (Figure 1D).

Visual stimuli spanned a wide range of spatiotemporal complexity. Four broad classes of stimuli were presented: (i) simple artificial stimuli such as full field flashes of white or black from gray and flashes of small Gabor patches; (ii) more complex artificial stimuli such as full field static or drifting gratings; (iii) naturalistic stimuli such as movie clips; and (iv) temporally shuffled natural movies. A no-stimulus condition consisting of a mean-luminance gray screen was also used (Figure 1E). Each stimulus class was displayed in multiple blocks in ∼3 h recording sessions.

Here, we quantify the temporal complexity of spiking activity throughout the session using the spectral differentiation metric of ND. Spike times are binarized in 5 ms bins and then convolved with a Gaussian window (10 ms SD, cut off at ± 25 ms; see section “Materials and methods”) to obtain the firing rate of individual neurons as a function of time, sampled at 200 Hz. The firing rate (FR) timeseries is then divided into non-overlapping 3 s windows *W*. Within each window, the FR is further typically split into 10 non-overlapping “state windows” with a length of *S* = 300 ms. The corresponding state vector is obtained by computing the power spectrum of each neuron’s activity within that state window and then concatenating the spectra of all neurons into a single vector. Differentiation at a given time is defined as the median Euclidean distance between all pairs of state vectors within the window *W* centered around that time (Figure 1F, see section “Materials and methods”).

Since ND is defined using the Euclidean distance, the metric is not additive, i.e., ND of activity for a combination of ensembles is not the sum of ND for individual ensembles. This is because Euclidean distance between two vectors is not the sum of distances in orthogonal subspaces. Thus, while ND primarily quantifies temporal complexity, it also accounts for the heterogeneity among neurons in a non-trivial manner. The metric does not, however, take into consideration spatial structure.

To enable comparison of ND across different ensembles of neurons, we apply additional normalizations (see section “Materials and methods” and Supplementary Figure 1-1). First, we note that if the firing rate of all neurons is scaled by a constant factor, the amplitude of the power spectrum, distances between pairs of spectra, and thus, ND for the ensemble, are all scaled by the square of that factor even though the temporal complexity is unchanged. Therefore, to account for potential differences in overall firing rates across ensembles, the firing rates are divided by the average firing rate of all neurons within the ensemble across the entire recording time. Second, each neuron contributes a fixed number of dimensions to the state space, equal to the length of the power spectrum (see section “Materials and methods”). To enable comparison across differently sized ensembles of neurons, we further divide ND by the square root of the number of neurons in the ensemble, to obtain a per-neuron normalized metric. These two normalizations account for the uncontrolled heterogeneity (number and mean firing rate of neurons) in the ensembles analyzed.

The length of the state window (state length or *S*; see section “Materials and methods”) determines the timescale at which neural activity is observed. In the next section, we report how differentiation changes with the state length.

### Dependence of ND on the temporal scale of observation

Before analyzing the neurophysiological results, we build intuition for the dependence of the spectral differentiation metric on the timescale of observation, by computing spectral differentiation for a signal using different values of state lengths *S*, ranging from 10 ms to 3 s, and lengths of windows, *W*, over which the number of states is quantified (3, 9, and 30 s). For all instances discussed below, the sampling rate of the signal is fixed at 200 Hz.

First, as a toy example, we use an artificial signal that takes random integer values drawn uniformly between 0 and 255, with a characteristic timescale of 600 ms (the value of the signal changes every 600 ms). As the state lengths increase above the characteristic timescale, variations in the signal within the state are averaged out, and the median pairwise distance between state vectors should decrease. For state lengths shorter than the characteristic timescale, the median distance between pairs of state vectors should remain constant. This intuition is borne out when we compute spectral differentiation (Figure 2A): it indeed remains constant for state lengths shorter than the characteristic timescale of the signal and decreases as a power law for longer state lengths. It is important to note that this trend can be different for a sparse signal (i.e., a signal which, for a given *W*, is mostly constant within the window): if the signal is sparse, then as *S* is shortened an increasing number of states have identical activity. Therefore, the median pairwise distance between state vectors continues decreasing to 0. Thus, for a sparse signal (such as spiking activity of neurons), spectral differentiation is expected to be optimal near the characteristic timescale of the signal and decrease for longer and shorter state lengths (Figure 2A).

As a second example, we survey the spectral differentiation of the visual stimulus itself, called stimulus differentiation (SD). We treat each pixel of the stimulus as a “neuron” and compute SD at different timescales, keeping *W* fixed at 3 s. Consistent with the non-sparse toy example above, for stimuli, SD remains constant up to the characteristic timescale (see section “Materials and methods”) and decreases with increasing state length as a power law beyond it (Figures 2B, C). These stimuli consist of signals that vary substantially over the entire length of the window, and thus, SD plateaus at state lengths shorter than the characteristic timescale. Two stimuli—flashes and short drifting gratings—are sparse stimuli, with activity concentrated within short intervals and constant mean-luminance gray outside of those intervals. For these two stimuli, SD has an optimum near the characteristic timescale and decreases for both shorter and longer state lengths, as expected.

Note that for a similar kind of activity (such as similar stimuli, or neural responses to similar stimuli), we expect the activity to occupy nearby regions in state space. Consequently, as the window size increases, the median distance between states should asymptotically approach a constant value, as we can observe in both SD and ND for an example experiment (Supplementary Figure 2-1). Therefore, for all of our analyses, we fix *W* at 3 s, a trade-off between longer windows where spectral differentiation is close to the asymptotic value and shorter windows that facilitate computational tractability.

Next, we ask how the ND of responses of individual neurons depends on the time scale. Consistent with the observations above, ND of activity of a large fraction of single neurons is maximized at some optimal timescale, corresponding to the characteristic timescale of their activity (Figures 2D, E; see section “Materials and methods”). Roughly 27% (373 / 1,429 per cortical area) of regular spiking (RS, putatively excitatory pyramidal) cortical neurons show such an optimal timescale (∼56% thalamic RS neurons), averaged across stimuli (Supplementary Figure 2-2). A slightly larger fraction (∼42%; 113 / 274 per area) of fast spiking (FS, putatively inhibitory interneurons) cortical neurons have an optimal timescale. A majority of the remaining neurons have an optimal timescale longer than 1 s, which was not probed, while a few have very low firing rates, and thus zero ND at all state lengths.

The intrinsic timescale of correlations of neural activity increases along the anatomical visual cortical hierarchy in this same dataset (Piasini et al., 2021). In comparison, the optimal timescale of ND for RS neurons is also shortest in the thalamic areas, and is longer in cortical areas; but surprisingly remains relatively constant (*p* > 0.05 for all stimuli, Supplementary Tables 2-1) across the visual cortical hierarchy. The optimal timescale is shortest in layer 4 for RS neurons (Supplementary Figure 2-2). These modulations with laminar depth or cortical area are largely restricted to RS neurons, and are not as strong in FS neurons (*p* > 0.05 with respect to hierarchy except for Gabor stimuli, Supplementary Table 2-2). Optimal timescales for FS neurons are around 40–60 ms shorter than for RS neurons. Overall, optimal timescales for individual neurons vary within a factor of 2 across stimuli, ranging from 100 to 225 ms in cortical area, and are not related to the optimal timescales for the corresponding stimulus differentiation.

Finally, we ask how ND for an ensemble of neurons depends on the timescale. Interestingly, although single neurons have an optimal ND timescale, this optimality is essentially lost for ensembles (Figure 2E and Supplementary Figures 2-4): ND increases at shorter and shorter timescales down to 10 ms (with a sampling rate of 200 Hz, we cannot consider state lengths shorter than 10 ms; since we do not find an optimal timescale within this range, we refer to this as ‘loss of optimality’ subsequently). Although individual neurons within the ensemble might have a similar characteristic timescale, the sparsity of firing and jitter or offsets in spike times are the likely causes for increasing ND at very short timescales. We verify this hypothesis by computing ND for an ensemble of virtual neurons, constructed by taking the spiking activity of a single neuron and adding random jitter or offset to the spike times. For neurons with a high firing rate, very small ensembles of 3–4 virtual neurons already show loss of optimality. For low firing rate neurons, the optimality of ND moves to shorter timescales as the number of virtual neurons increases (Supplementary Figure 2-3) but remains above 10 ms. Thus, with an increasing number of neurons in an ensemble, sparsity of their collective activity is reduced, resulting in the decrease of the optimal times for ND of the ensemble activity.

Analysis of our empirical data shows that even for a small number of neurons, the ND optima shift into the sub-10 ms range. This underscores the important point that ND should not necessarily be expected to be maximal at a timescale relevant to the stimulus or the experience, unlike a quantity that would fully characterize conscious experience, which involves both differentiation of the experience and integration of its components (Tononi et al., 2016). Rather, ND should be considered a relative measure defined at a timescale chosen by the observer, which can be compared across conditions (stimuli, brain states, etc.) or, after using an appropriate normalization, across neural ensembles. The dependence of ND on the timescale of observation is affected by the characteristic timescale of the underlying signal, which can have significant consequences on the interpretation of differentiation results. For example, at short timescales, the stimulus differentiation of static gratings is higher compared to natural movies, but at longer timescales, the relationship inverts (Figure 2F).

In the following sections, we study the dependence of ND on cortical areas, layers, and the complexity or relevance of the stimuli. Subjective experience in humans is thought to occur on a timescale of approximately 300 ms (Del Cul et al., 2007; Herzog et al., 2016, 2020), and therefore, we restrict all further analysis to this timescale (*S* = 300 ms).

### Modulation of ND by different visual stimuli across brain areas

**FIGURE 3:**
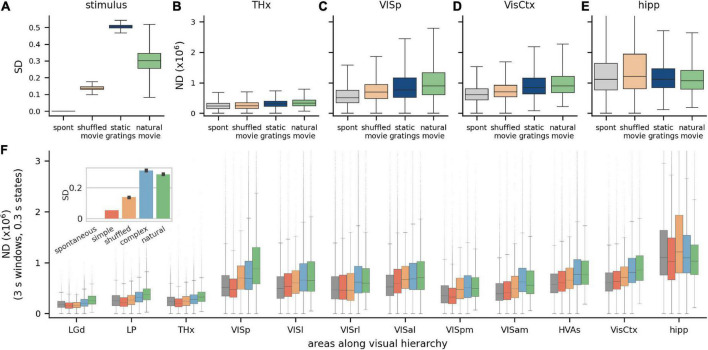
Modulation of ND by visual stimuli across brain areas. **(A)** Stimulus differentiation (SD) for the four stimuli, computed for *S* = 300 ms, for stimulus pixels and **(B–E)** neurophysiological differentiation (ND) (×10^6^) for 4 neuronal ensembles; these do not simply reflect SD but putative relevance of stimulus—static grating stimuli (blue) are more differentiated than natural movie stimuli (green), but responses to movies are more differentiated than to gratings in most brain areas (all pairwise differences are statistically significant). THx, all visual thalamic areas; VisCtx, all cortical visual areas; hipp, hippocampal areas **(F)**. ND increases going from thalamus to VISp but remains constant over the visual cortical hierarchy. Simple artificial stimuli (full screen flashes and Gabor patch flashes) evoke least ND, followed by complex artificial stimuli (gratings) and natural movies (green) typically evoke the highest ND in most areas. Interestingly, (a) time-shuffled movies sometimes evoke a higher or comparable ND as natural movies and (b) spontaneous activity sometimes evokes a higher ND than simple artificial stimuli. Hippocampal ND is not correlated with the stimulus type, consistent with it not playing a direct role in vision. Inset: SD for the five stimulus classes.

The Visual Coding Neuropixels dataset includes a wide variety of images and movies, with varying levels of stimulus differentiation (SD), with gray screen having zero differentiation, static gratings having the highest and natural movies being intermediate (for the 300 ms state length; Figure 3A). In addition to SD, another factor that could influence the ND of responses is the relevance or meaningfulness of the stimulus to the animal. In a brain region whose activity determines perceptual experience, ND is expected to have a stronger correlation with meaningfulness of the stimulus than with SD. Although we cannot ascertain the actual meaningfulness of the stimuli in this dataset to the mouse, we can ascribe a putative meaningfulness or relevance in terms of how close the stimuli are to a naturalistic setting. For example, an unchanging gray screen is putatively the least relevant; artificial stimuli unlikely to occur in a natural setting, such as gratings, are somewhat more relevant; and naturalistic movies are likely to have the most relevance to the animal, given the higher order spatiotemporal structure in movies that is absent in gratings.

We compare the ND of neural responses to these stimuli in 4 regions: visual thalamus (THx, Figure 3B), primary visual cortex (VISp, Figure 3C), all visual cortices taken together as an ensemble (VisCtx, Figure 3D) and, as a control, the hippocampus (hipp, Figure 3E), which is not a visual region (due to the large number of neurons recorded, ND is statistically significantly different between all pairs of stimuli with *p* < 0.001; pairwise Games-Howell test for each region). Consistent with expectation, the response to gray screen (spontaneous activity) is least differentiated in all visual brain areas. This is not surprising given that it also has zero SD. Interestingly, however, even though static gratings are much more differentiated than natural movies (Figure 3A), responses to gratings are less differentiated than those to movies in all three visual brain areas shown. Thus, the ND metric does not simply reflect the differentiation of the stimulus but correlates with the putative relevance of the stimulus to the mouse.

Next, we group the Visual Coding dataset into 5 broad categories with increasing putative meaningfulness—no stimulus (gray screen or spontaneous activity), simple artificial stimuli (full screen flashes and flashes of small Gabor patches), time-shuffled movies, complex artificial stimuli (static and drifting gratings) and natural stimuli (movies with higher order spatiotemporal correlations). SD for these stimuli is ordered differently than their putative relevance: spontaneous, simple artificial, shuffled, natural and finally complex artificial (Figure 3F, inset). This raises an interesting question of whether ND follows either of the two orderings (putative relevance or SD) or is entirely different. We expect ND to reflect putative relevance for ensembles encoding stimulus meaningfulness, and to reflect SD for ensembles encoding stimulus information in general. For areas not involved in vision, such as the hippocampus, we do not expect any particular ordering of ND.

We compute ND for ensembles of neurons restricted to individual areas along the visual hierarchy, or their combinations, such as neurons from all higher visual areas (HVAs), or the entire visual cortex (VisCtx), etc. (as noted earlier, ND is not an additive metric; ND of a combination of areas is not the same as the sum of ND from individual areas). Broadly, we find that ND increases going from thalamic areas to the cortex, but mean ND remains roughly constant over the hierarchy of visual cortical areas (Figure 3F; *p* > 0.05 for all but natural stimuli, Supplementary Table 3-1).

Across much of the visual hierarchy, putatively less meaningful stimuli (gray screen and simple artificial stimuli) evoke the least differentiated responses, complex artificial stimuli evoke more, and natural stimuli evoke the most differentiated responses (exceptions are VISrl and VISam, in which ND follows the same ordering as SD). The latter point is particularly interesting, since SD is higher for complex artificial than natural stimuli. The ND metric may therefore reflect the putative relevance of the stimuli rather than the pixel-wise stimulus differentiation in a majority of individual visual areas.

Note that the no-stimulus condition has a more differentiated response than simple artificial stimuli in a few areas (THx, VISp, and VISpm). This observation is also consistent with the possibility that ND reflects a measure of perceptual experience rather than just the stimulus properties (see section “Discussion”).

Finally, the hippocampus exhibits substantially higher ND than the other areas analyzed. This suggests a greater diversity of activity states in the hippocampus than in the visual cortex and thalamus, possibly reflecting a more narrow, specialized role of the latter structures dedicated primarily to visual processing. This does not mean, however, that the hippocampus is necessarily more involved in perception—only that the activity there is more temporally complex (i.e., occupies a larger volume of the state space). For identifying areas involved in conscious perception, it is not the magnitude of responses, but correlation of responses with meaningfulness, that is relevant. If the ND of activity in a region is specifically correlated with putative meaningfulness, then activity within that region can be said to reflect percepts. Conversely, if activity in a region reflects percepts, its ND will not only be significantly modulated by stimulus type, but will be correlated with putative meaningfulness. Our data indicates that ND of hippocampal activity is significantly modulated by stimulus type, but it is not correlated with putative meaningfulness. This is consistent with our understanding that the hippocampus is not directly involved in perception.

Neural ensembles that represent subjective perception change their activity state with changing percepts. Since percepts are observed to change over a timescale of 300 ms (at least in humans), we have examined how ND depends on the stimulus meaningfulness using 300 ms states. However, this also means that for times shorter than 300 ms, the activity states need not correlate with meaningfulness. We verify this hypothesis by computing ND for 300 ms windows (instead of 3 s), and 30 ms states (instead of 300 ms). We find that indeed in this case, ND is no longer correlated with putative meaningfulness in any of the visual areas but instead ND becomes strongly correlated with SD (Supplementary Figure 3-1). Thus on such short timescales, complexity of activity in all visual areas seem to be driven by the complexity of the stimulus. Only over longer timescales, the complexity of activity reflects the complexity of percepts.

We performed a similar analysis using a simpler metric of differentiation of neural activity: the variance of mean firing rate across states (see section “Materials and methods” for definition, here abbreviated as mfrD). We observe that unlike spectral differentiation, which follows the ordering of putative meaningfulness in several individual areas, and especially in the higher visual areas/entire cortex, mfrD simply follows the ordering of stimulus differentiation in all areas (including the aggregate HVAs and VisCtx, Supplementary Figure 3-2). This suggests that the spectral differentiation metric is potentially more relevant for assessing subjective meaningfulness than canonical measures of variability of brain states.

While the results presented in this section are obtained for regular spiking neurons only, they hold for fast spiking neurons as well (Supplementary Figure 3-3). Although ND of fast and regular spiking neurons shows similar patterns, the overall magnitude of ND of cortical FS neurons is about half that of RS neurons, while thalamic FS neurons have similar ND as RS neurons. Given the similarities between ND of RS and FS neurons, we restrict our analysis to RS neurons in subsequent sections.

### Modulation of ND by visual stimuli across cortical layers

**FIGURE 4:**
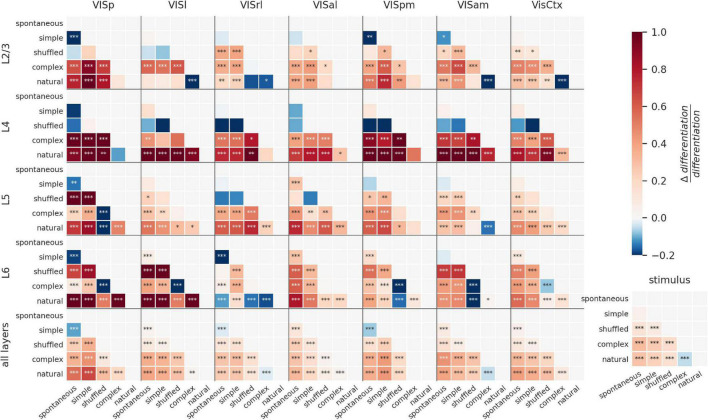
Modulation of ND by visual stimuli across cortical layers. Difference in ND of cellular responses to stimulus pairs for different areas and layers. Within each panel, each square shows difference of ND for y-axis and x-axis stimuli (red indicates y-x is positive; blue indicates y-x is negative). Stars indicate statistically significant differences (*p < 0.01; ^**^p < 0.001; ^***^p < 0.0001). L6 shows the most significant modulation of ND by stimulus, although most other areas also show significant modulation for several stimulus pairs. ND is consistent with putative meaningfulness in layers 4 and 5; but in layers 2/3 and 6, natural movies often evoke lower ND and shuffled movies evoke higher ND than other stimuli. This difference disappears when the analysis is restricted to when the mouse is running (Supplementary Figure 4-1).

To visualize the modulation of ND by visual stimuli for all areas and layers, we plot the difference of ND for all pairs of stimuli as a matrix (Figure 4). The stimuli are ordered along the axes according to their putative relevance, so that positive differences (in red) indicate a positive relationship between the putative relevance and evoked ND (stars indicate significance: *p < 0.01; ^**^p < 0.001; ^***^p < 0.0001). Pairwise differences and significance are obtained by fitting a linear mixed effects model with fixed effects of layer, area, and stimulus category, and random effect of mouse. Consistent with the previous section, this visualization reveals ND of entire areas is positively correlated with putative relevance for all areas except VISrl and VISam (Figure 4, bottom row, bottom-right cell: natural—complex artificial). Also as previously noted for entire areas (i.e., combining all layers), spontaneous activity is more differentiated than the responses to simple artificial stimuli in VISp, VISrl and VISpm (Figure 4, bottom row, top-left cell).

We next investigate the layer-specific contributions to the ND of responses to different stimuli. Overall, within layers 4 and 5 of individual areas, differences in ND for most pairs of stimuli are broadly consistent with putative meaningfulness. Interestingly, ND of responses of layers 2/3 and 6 of higher visual areas does not follow this pattern, and natural movies tend to evoke lower ND compared to several other stimuli; shuffled movies tend to evoke higher ND than natural or complex artificial stimuli (see section “Discussion”).

Depending on the behavioral state of the animal, the differences between layers become starker (Supplementary Figure 4-1). When the animal is running, the discrepancies between ND and putative meaningfulness in layer 6 are highly reduced compared to the resting state. Secondly, the overall statistical significance of modulation of ND by stimuli is much reduced in all layers except layer 6 in the running state.

Note that layers 2/3, 4, 5, and 6 have a comparable number of recorded neurons (52 ± 25, 44 ± 17, 73 ± 26 and 52 ± 24, respectively on average per mouse) while layer 1 has fewer (15 ± 9) and a more inconsistent number of neurons across experiments. Layer 1 is thus excluded from this analysis.

ND patterns in individual areas can be very different from those seen when the areas are combined. For example, shuffled movies evoke higher ND than complex artificial stimuli in layer 6 of all individual cortical visual areas (VISp, VISl, VISpm, VISam are significant). Yet, when all neurons are combined (L6 AllVis / HVAs), their ND is approximately equal for responses to complex artificial and shuffled stimuli (no significant difference), underscoring the non-additive nature of the ND metric.

Together, this shows that the differences between stimuli are reflected in ND of neural activity in all layers individually. However, the evoked ND is not necessarily consistent with putative relevance of the stimuli, as seen in layers 2/3 and 6. When all layers are taken together, however, the evoked ND is consistent with putative relevance, suggesting that it’s the larger ensembles of neurons across all layers that may be supporting or reflecting stimulus meaningfulness.

### ND during behavior

Although we show a correlation between the putative relevance of stimuli and ND of neural response, we cannot directly access the actual experience of the mouse—i.e., whether it even perceives the stimulus or not. Towards addressing this concern, we compute ND of responses to visual stimuli as the mouse performs a visual behavior task (Siegle et al., 2021).

**FIGURE 5:**
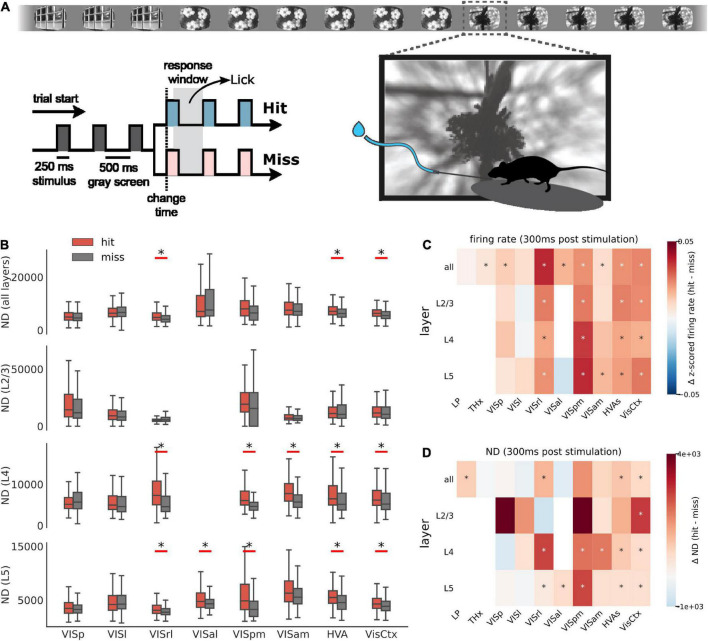
ND during behavior. **(A)** Schematic of the change detection task. A series of images is flashed in front of a water-deprived mouse. On detecting a change in the identity of the image, the mouse is trained to lick the spout and is rewarded with water (hit). An undetected change is called a miss. **(B)** ND of first 300 ms of spiking activity post image presentation for hit (red) and miss trials (gray) across areas and layers. Aggregate areas (all higher visual areas or all visual cortical areas) show a significant difference in ND for hits vs. misses, but not individual areas (Benjamini–Hochberg corrected with α = 0.01). Data from single experiment. Summary across all experiments for firing rate **(C)** and ND **(D)** differences between hit and miss. Stars indicate statistically significant differences (Benjamini–Hochberg corrected with α = 0.01). Hits are associated with elevated firing rate in several areas. In contrast, increase in ND is more specific to aggregate areas.

In this task, water-deprived mice (*n* = 24) were head-fixed but otherwise free to run on a disk. A series of images was flashed in front of the mice, and the mice were trained to detect and respond to a change in the identity of the image by licking at a spout. Successful detection of change, called a hit, was rewarded with water. The mice did not get the water reward if they did not lick in response to the image change, called a miss (Figure 5A). Neural activity was recorded using Neuropixels probes, yielding a total of 7,638 neurons (318 ± 150 per mouse) with SNR > 2.5. For a mouse detecting an image change and responding, hits are likely correlated with successful perception and misses with lack of perception. We thus expect ND of spiking activity to be higher for hits compared to misses in areas representing experienced percepts.

Indeed, in aggregate ensembles, like all higher visual area neurons (HVAs) or all visual cortical neurons (VisCtx), hits are associated with a more differentiated response in the first 300 ms post stimulus compared to misses (Figure 5B; significance determined after applying the Benjamini–Hochberg correction for multiple corrections with α = 0.01). This difference is statistically significant irrespective of the layer. The 300 ms window is chosen as a trade-off between using the shortest time post-stimulation and including sufficient number of state windows to compute ND (it also includes the reaction time of the mice). We use state lengths of 60 ms instead of 300 ms for a total of 5 states (300 ms total window length) over which ND is computed. In contrast to the aggregate areas, individual areas typically do not show a significant difference in ND for hits compared to misses. Note that for fair comparison, we subsampled units from aggregate areas to match the mean number of units in individual areas before computing ND.

In general, hit trials are associated with elevated overall firing rates (Figure 5C). The increase of firing rates during hits is observed in several individual cortical areas and layers, and in aggregated areas as well. However, unlike the firing rate, difference in ND for hits and misses appears largely in aggregate areas and is not common in individual areas (Figure 5D). ND across regions is thus not a mere reflection of firing activity but appears to be consistent with perception.

## Discussion

Differentiation of neural activity (ND) at the whole-brain scale in response to subjectively meaningful and meaningless visual stimuli has been studied in humans with non-invasive modalities such as fMRI and EEG (Boly et al., 2015; Mensen et al., 2017, 2018) that limit the spatiotemporal resolution of observation of neural activity. This is the first study to characterize the spectral differentiation metric at the cellular level and at the millisecond timescale, for ensembles spanning across multiple visual cortical areas within the mouse brain. Overall, we see that ND of activity in many visual brain regions, and especially at the scale of the entire visual cortex, does not simply reflect stimulus differentiation, but is correlated with the putative meaningfulness of stimuli, consistent with human studies (Figure 3).

Although putatively more meaningful stimuli generally evoked more differentiated neural responses, our analysis revealed a curious exception: in some areas, the no-stimulus condition, corresponding to a gray screen, had higher ND compared to simple artificial stimuli like flashes and Gabor patches. It is well known that even in the absence of stimuli, structured patterns of activity are spontaneously generated in the brain, often correlated with behavior (Ringach, 2009; Stringer et al., 2019). It is possible that these patterns are more differentiated than the activity evoked by strong but temporally simple external stimuli like full-field flashes that may strongly constrain activity patterns in the visual system. Temporally more complex inputs (such as gratings or natural movies), on the other hand, seem to drive activity across more varied states compared to spontaneous patterns in all the brain regions we investigated. Furthermore, when the mouse was running, simple artificial stimuli evoked a more differentiated response compared to spontaneous activity. This could be because activity in the visual areas is more strongly driven by visual inputs in the running state and spontaneous fluctuations are suppressed (Dadarlat and Stryker, 2017), consistent with the interpretation of ND as capturing more than the passive representation of external information.

ND of activity of entire areas generally tracked the putative meaningfulness of stimuli. However, this was not true within individual layers. Though ND was significantly modulated by stimuli in all layers, the ND patterns in layers 2/3 and 6 were very different from those of other layers or the entire areas; especially with respect to the comparison between complex artificial, natural, and shuffled stimuli. In the behavioral study, we see that ND in individual areas was not significantly different for hit and miss trials, but when combined, the aggregate of all higher visual areas showed significantly higher ND for hits compared to misses (Figure 5). Together these observations suggest that the activity in the visual cortex as a whole might be more correlated with meaningfulness than in its individual parts. Our current understanding of the visual cortical hierarchy suggests that more complex objects or concepts are represented higher up in the hierarchy. This might lead to the hypothesis that ND should also become more correlated with meaningfulness as we go higher up the visual hierarchy. However, it is important here to make the distinction between representation of information and subjective perception. Our results suggest that activity within individual areas, even high up in the hierarchy, is not as correlated with subjective perception as the overall activity of the visual areas combined.

In the canonical cortical microcircuit, feedforward information propagates upwards from L2/3 (Bastos et al., 2012), while L5 pyramidal (L5p) cells are involved in integrating feedback and feedforward streams of information, mediated by thalamic inputs (Aru et al., 2020; Suzuki and Larkum, 2020), and propagating outputs to other cortical and subcortical areas. Recent work suggests that such dendritic integration of information by L5p cells is required for experiencing specific contents of consciousness (Takahashi et al., 2016). Consistent with this picture, in our study also, we observe that ND is correlated with putative meaningfulness in deep layers that integrate feedback and feedforward information, mediated by the thalamus; but not in layer 2/3, which may be related to the larger role of this layer in relaying feedforward information to higher areas.

In a passive viewing paradigm, it is not possible to ascertain the ground truth regarding the perception of animals. To address this, we analyzed ND in a behavioral paradigm, where there is higher confidence regarding the perception of animals (Figure 5). The mice might be employing different strategies in the change detection task, some of which may not involve perception of the images: for example, they could learn the average time between image changes and lick randomly around those times. For such strategies, we do not expect any difference in ND for hits or misses. Yet, we find that hits correspond to significantly higher ND in aggregate areas (HVAs or the entire visual cortex) compared to misses, consistent with the presumed strategy of making a decision based on a percept: perceived image changes lead to hits and unperceived ones to misses. Moreover, even though firing rates are elevated for hits in several cortical areas, differences in ND were restricted to aggregate areas, reflecting the specificity of the ND metric.

The notion of differentiation is grounded in the observation that there must be a one-to-one mapping between subjective perception and states of its neural substrate. Temporal differentiation of activity is also postulated by the integrated information theory of consciousness (IIT) as a necessity for conscious experience, in addition to integration (activity in different parts or at different times should not be completely independent) (Sarasso et al., 2014; Koch et al., 2016; Tononi et al., 2016; Ruiz de Miras et al., 2019). According to this theory, a combined measure of integration and differentiation, integrated information, is expected to be maximal at the spatiotemporal scale of subjective experience (Tononi et al., 2016). As we observe, the differentiation (without integration) of ensemble activity does not have an optimal timescale, but we do find an optimal timescale of ∼100 ms for single neuron differentiation (Figure 2).

In conclusion, we find that the spectral differentiation of activity can be a useful tool to identify specific subpopulations of neurons that may be involved in subjective perception. These results, in conjunction with human studies, where ND was found to correlate with subjective reports of meaningfulness, reflect the future potential to objectively infer the quantity of experience of subjects who otherwise have no ability to report it, such as animals or humans with disorders of consciousness.

## Data availability statement

The original contributions presented in this study are included in the article/Supplementary material, further inquiries can be directed to the corresponding authors.

## Ethics statement

The animal study was reviewed and approved by Allen Institute’s Institutional Animal Care and Use Committee.

## Author contributions

SRG, WGPM, WM, YB, SO, GT, CK, and AA designed the research. SG, WGPM, WM, CK, and AA analyzed the data. SG, CK, and AA wrote the manuscript. All authors contributed to the article and approved the submitted version.
